# Ultra–low-contrast transfemoral TAVI using the MyVal transcatheter heart valve: a prospective single-center feasibility and safety study

**DOI:** 10.3389/fcvm.2026.1878620

**Published:** 2026-08-26

**Authors:** Georgios E. Papadopoulos, Ilias Ninios, Maria Kalaitzoglou, Sotirios Evangelou, Andreas Ioannides, Athinodoros Nikitopoulos, Georgios Bompas, Vlasis Ninios, Grigorios Giamouzis

**Affiliations:** 1 Cardiology Department, Interbalkan Medical Center, Thessaloniki, Greece; 2 Department of Cardiology, University Hospital of Larissa, Faculty of Medicine, University of Thessaly, Larissa, Greece

**Keywords:** acute kidney injury, aortic stenosis, balloon-expandable valve, chronic kidney disease, MyVal, renal function, transcatheter aortic valve implantation, ultra–low contrast

## Abstract

**Background:**

Acute kidney injury remains clinically relevant after transcatheter aortic valve implantation (TAVI), particularly in chronic kidney disease, and iodinated contrast is a modifiable procedural contributor.

**Methods:**

We conducted a prospective single-center study of 50 patients undergoing transfemoral TAVI with an intraprocedural ultra–low-contrast strategy using the MyVal transcatheter heart valve. The primary feasibility endpoint was successful TAVI completion with total intraprocedural contrast volume ≤10 mL. Procedural outcomes, valve performance, renal function, and 30-day clinical events were assessed. Contrast-enhanced cardiac CT angiography was used for planning. Median CT contrast volume was 45 mL; median CT-to-TAVI interval was 18 days. Exploratory 6-month renal and clinical follow-up was analyzed when available.

**Results:**

Ultra–low-contrast MyVal TAVI was completed in all patients, with median contrast volume 8 mL [interquartile range, 6–10] and no procedure exceeding 10 mL. Baseline renal impairment was frequent, with median estimated glomerular filtration rate 38 mL/min/1.73 m^2^ and 15 patients having estimated glomerular filtration rate <30 mL/min/1.73 m^2^. Technical success was achieved in 50 patients, with no intraprocedural death, coronary obstruction, annular rupture, valve embolization, second valve implantation, or emergency surgery. Valve hemodynamics improved, with mean aortic gradient decreasing from 45.2 ± 11.8 mmHg at baseline to 8.7 ± 3.2 mmHg at 30 days, and no moderate or severe paravalvular regurgitation. Renal function remained stable, with no significant creatinine increase and modest estimated glomerular filtration rate increase at 30 days. Acute kidney injury occurred in 1 patient and was limited to stage 1; no patient developed stage 2 or 3 acute kidney injury or required new renal replacement therapy. Among patients with 6-month laboratory follow-up, renal function remained stable or improved, and no patient initiated renal replacement therapy.

**Conclusions:**

In this single-center feasibility study, an intraprocedural ultra–low-contrast transfemoral TAVI strategy using the MyVal valve was feasible, with preserved early procedural success and valve performance. Renal function remained stable through 30 days, and AKI was infrequent; however, without a comparator group, these findings should be interpreted as descriptive feasibility and early safety observations rather than evidence of renal protection, AKI reduction, or superiority over standard contrast-guided TAVI. Larger comparative studies are required.

## 1 Introduction

Transcatheter aortic valve implantation (TAVI) has become an established treatment for patients with severe symptomatic aortic stenosis across a broad spectrum of surgical risk, supported by progressive improvements in patient selection, procedural planning, device technology, and periprocedural management (1, 2). As TAVI expands to older, frailer, and increasingly comorbid populations, procedural optimization has shifted beyond valve implantation alone toward the prevention of avoidable complications that may influence early recovery and longer-term outcomes (1–3). Among these, acute kidney injury (AKI) remains clinically relevant, particularly in patients with pre-existing chronic kidney disease (CKD), who represent a substantial proportion of contemporary TAVI candidates and are more vulnerable to periprocedural renal deterioration (4, 5).

AKI after TAVI is multifactorial and may result from baseline renal vulnerability, hemodynamic instability, rapid pacing, bleeding, vascular complications, atheroembolism, anemia, inflammation, and exposure to iodinated contrast media (4–6). Although the relative contribution of contrast administration may vary among individual patients, contrast exposure remains one of the few modifiable procedural factors. Several studies have shown that contrast burden adjusted for renal function, particularly the contrast volume-to-eGFR or contrast volume-to-creatinine clearance ratio, is associated with post-TAVI AKI and adverse early outcomes (6–8). This has provided the rationale for renal function–adapted contrast dosing and for the development of contrast-sparing TAVI strategies in patients at increased renal risk (6–9).

In this context, minimalist, low-contrast, ultra–low-contrast, and zero-contrast TAVI approaches have emerged as potential strategies to reduce renal injury while preserving procedural safety. Early prospective and observational experiences suggest that TAVI can be performed with markedly reduced or absent iodinated contrast exposure in selected patients with CKD, with acceptable procedural success and early clinical outcomes (9, 10). However, most available reports remain limited by small sample size, heterogeneous imaging workflows, mixed valve platforms, and variable definitions of contrast minimization. Therefore, additional prospective data are needed to clarify whether an ultra–low-contrast strategy can be implemented reproducibly without compromising valve positioning, hemodynamic performance, paravalvular regurgitation, or early safety (3, 9, 10).

The MyVal transcatheter heart valve is a balloon-expandable platform with an expanding body of clinical evidence in native aortic stenosis (11, 12). Initial clinical experience demonstrated favorable procedural and echocardiographic outcomes with MyVal in patients with severe symptomatic aortic stenosis (11). More recently, randomized data have supported the non-inferiority of the MyVal THV series compared with contemporary transcatheter heart valves for early clinical endpoints (12). Nevertheless, data specifically evaluating an ultra–low-contrast TAVI strategy using exclusively the MyVal platform remain limited.

Accordingly, the present prospective single-center study evaluated the feasibility, safety, and early outcomes of an ultra–low-contrast transfemoral TAVI strategy using exclusively the MyVal transcatheter heart valve. We specifically assessed procedural success, contrast burden, renal-function trajectory, AKI, early clinical outcomes, valve hemodynamics, and paravalvular regurgitation through 30-day follow-up, with additional exploratory analyses according to baseline renal function, contrast volume, contrast volume/eGFR ratio, MyVal valve size category, annular dimensions, and chronological procedural experience.

## 2 Materials and methods

### 2.1 Study design and population

This was a prospective, single-center observational feasibility study evaluating an ultra–low-contrast transfemoral TAVI strategy using exclusively the MyVal transcatheter heart valve system. The study cohort consisted of consecutive patients undergoing attempted native-valve transfemoral MyVal TAVI using the ultra–low-contrast protocol at the Cardiology Department of Interbalkan Medical Center, Thessaloniki, Greece, between January 2023 and January 2026.

Eligible patients were adults with severe symptomatic native aortic stenosis who were accepted for transfemoral TAVI after multidisciplinary Heart Team evaluation and in whom implantation with the MyVal balloon-expandable transcatheter heart valve was planned. Severe aortic stenosis was diagnosed according to contemporary echocardiographic criteria, integrating aortic valve area, indexed aortic valve area, mean transvalvular gradient, peak aortic velocity, left ventricular function, flow status, and clinical symptoms. The decision to proceed with TAVI rather than surgical aortic valve replacement was made by the institutional Heart Team according to age, surgical risk, comorbidity burden, frailty, anatomical suitability, and patient preference.

Patients were included irrespective of baseline renal function. The study cohort consisted of consecutive patients undergoing attempted native-valve transfemoral TAVI with the MyVal balloon-expandable transcatheter heart valve using the ultra–low-contrast protocol. No additional anatomical exclusion criteria specific to the ultra–low-contrast protocol were prespecified once native-valve transfemoral MyVal TAVI had been selected after multidisciplinary Heart Team assessment and pre-procedural imaging review. In particular, bicuspid valve morphology, low coronary height, severe aortic-valve calcification, horizontal aorta, peripheral artery disease, and severe iliofemoral calcification or tortuosity were not considered automatic exclusions. Patients were excluded if they underwent non-transfemoral TAVI, valve-in-valve TAVI, TAVI with a valve platform other than MyVal, emergency TAVI for cardiogenic shock, or if essential baseline or follow-up renal-function data were unavailable. Patients receiving chronic renal replacement therapy before TAVI were not excluded by protocol but were analyzed separately if present. In the present cohort, no patient was receiving chronic dialysis before the procedure.

The study was designed as a single-arm feasibility and safety study. No contemporaneous or historical control group was used.

### 2.2 Ethics statement

The study was conducted in accordance with the principles of the Declaration of Helsinki and was approved by the institutional ethics committee. All patients provided written informed consent for the TAVI procedure and for the prospective collection and analysis of anonymized clinical, imaging, procedural, and follow-up data. The study was reported in accordance with the principles of transparent reporting for observational clinical research.

### 2.3 Pre-procedural assessment

All patients underwent standardized pre-procedural clinical, laboratory, electrocardiographic, echocardiographic, and anatomical assessment before TAVI. Baseline clinical variables included age, sex, body mass index, cardiovascular risk factors, relevant comorbidities, previous coronary revascularization, atrial fibrillation, peripheral artery disease, chronic obstructive pulmonary disease, prior stroke or transient ischemic attack, anemia, New York Heart Association functional class, and surgical-risk scores, including EuroSCORE II and STS-PROM.

Baseline laboratory assessment included serum creatinine, estimated glomerular filtration rate, hemoglobin, and routine biochemical and hematological parameters. Estimated glomerular filtration rate was calculated using the 2021 CKD-EPI creatinine equation and expressed as mL/min/1.73 m^2^. Chronic kidney disease categories were defined according to baseline eGFR. For the main renal subgroup analysis, patients were stratified into those with eGFR ≥30 mL/min/1.73 m^2^ and those with eGFR <30 mL/min/1.73 m^2^. Additional descriptive categories included eGFR ≥60, 45–59, 30–44, 15–29, and <15 mL/min/1.73 m^2^.

Transthoracic echocardiography was performed before TAVI to assess aortic stenosis severity, left ventricular ejection fraction, aortic valve area, indexed aortic valve area, mean transvalvular gradient, peak aortic velocity, concomitant valvular disease, and pulmonary artery systolic pressure. Aortic stenosis severity was adjudicated using an integrative approach, particularly in patients with low-flow or low-gradient patterns.

Anatomical assessment included evaluation of aortic valve morphology, annular dimensions, aortic root anatomy, left ventricular outflow tract calcification, coronary heights, sinus of Valsalva dimensions, sinotubular junction dimensions, annular eccentricity, aortic valve calcification burden, aortic angulation, and iliofemoral access suitability. Variables collected for the anatomical analysis included annular area, annular perimeter, annular eccentricity index, aortic valve calcium score, presence of moderate or severe LVOT calcification, left main and right coronary heights, horizontal aorta, minimal iliofemoral luminal diameter, severe iliofemoral calcification, and severe iliofemoral tortuosity.

### 2.4 CT reconstruction and fluoroscopic projection planning

All patients underwent pre-procedural cardiac CT angiography with multiplanar reconstruction. CT analysis included annular area, perimeter, diameters, eccentricity, left ventricular outflow tract anatomy and calcification, aortic-valve calcium burden, coronary heights, sinus of Valsalva dimensions, sinotubular-junction dimensions, aortic angulation, and iliofemoral access anatomy. CT reconstruction was also used to identify the intended coplanar fluoroscopic deployment projection before the procedure.

The term “ultra–low contrast” referred specifically to intraprocedural iodinated contrast exposure during TAVI. It did not refer to a fully contrast-free pre-procedural planning pathway. Standard contrast-enhanced cardiac CT angiography was performed in all patients. The volume of iodinated contrast administered during CT angiography and the interval between CT angiography and TAVI were recorded retrospectively from the imaging records. Pre-procedural CT contrast volume was reported separately and was not included in the primary intraprocedural feasibility endpoint.

The intraprocedural working projection was the right–left coronary cusp overlap view. In this projection, the right and left coronary cusps are superimposed, allowing the non-coronary cusp to be isolated and used as a reproducible fluoroscopic reference for the annular plane. The intended projection was planned from the pre-procedural CT reconstruction and verified fluoroscopically at the beginning of the procedure.

### 2.5 Ultra–low-contrast strategy

The transfemoral TAVI procedure was performed under conscious sedation in the Hybrid Operating Room of the Interbalkan Medical Center, utilizing the MyVal platform by a single operator. Femoral artery access was obtained through surgical cut-down technique by the same vascular surgeon.

Secondary arterial access was obtained via the left or right radial artery and a 5F pigtail catheter was advanced to the aortic root and positioned at the level of non-coronary cusp. In the right–left coronary cusp overlap projection, the distal pigtail loop was aligned fluoroscopically at the annular plane and served as the procedural “zero level” for MyVal valve landing. The aortic valve was then crossed in standard fashion, and a stiff guidewire was positioned in the left ventricle. Balloon aortic valvuloplasty was performed selectively according to valve morphology, calcification burden, crossing characteristics, and operator judgment.

**Figure 1:**
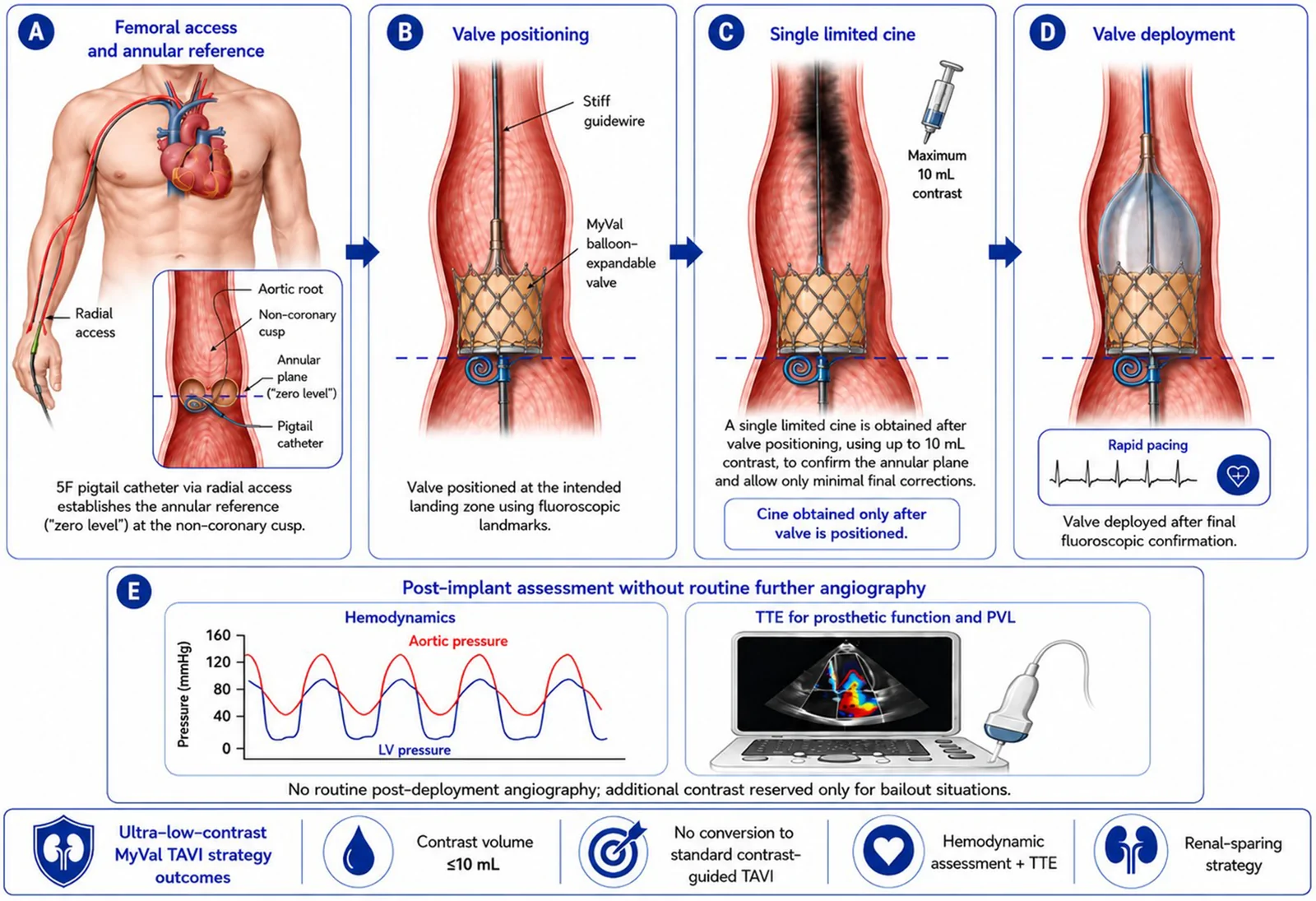
Ultra–low-contrast MyVal TAVI procedural strategy. Schematic overview of the ultra–low-contrast transfemoral TAVI workflow using the MyVal balloon-expandable transcatheter heart valve. **(A)** Femoral access for valve delivery and radial access for a 5F pigtail catheter positioned in the non-coronary cusp. In the right–left coronary cusp overlap projection, the distal pigtail loop is aligned with the annular plane and serves as the procedural “zero level.” **(B)** The MyVal prosthesis is advanced over a stiff left ventricular guidewire and positioned fluoroscopically relative to the pigtail-defined annular reference. **(C)** After initial positioning, a single limited aortographic cine acquisition using ≤10 mL of iodinated contrast is performed to confirm the annular plane, prosthesis depth, and coaxiality and to permit minor final adjustments. **(D)** The valve is deployed under rapid ventricular pacing after final fluoroscopic confirmation. **(E)** Post-implant assessment is performed using fluoroscopy, invasive hemodynamics, and transthoracic echocardiography to evaluate prosthetic valve function and paravalvular regurgitation, without routine post-deployment angiography. Additional contrast administration is reserved for bailout or safety-critical situations. MyVal, MyVal balloon-expandable transcatheter heart valve; PVL, paravalvular leak; TAVI, transcatheter aortic valve implantation; TTE, transthoracic echocardiography.

The MyVal transcatheter heart valve was advanced across the native aortic valve and positioned under fluoroscopic guidance in the right–left coronary cusp overlap projection, using the pigtail-defined annular plane as the principal reference for implantation depth. Once the prosthesis was positioned at the intended landing zone, a single limited aortographic cine acquisition was performed in the same projection using up to 10 mL of iodinated contrast. This acquisition was used to confirm the pigtail position at the annular plane and the relationship between the MyVal prosthesis and the intended landing zone, allowing only minimal final adjustments in prosthesis depth or coaxiality before deployment. No additional routine angiographic acquisitions were planned. The procedural workflow is illustrated in Figure 1.

**Figure 2:**
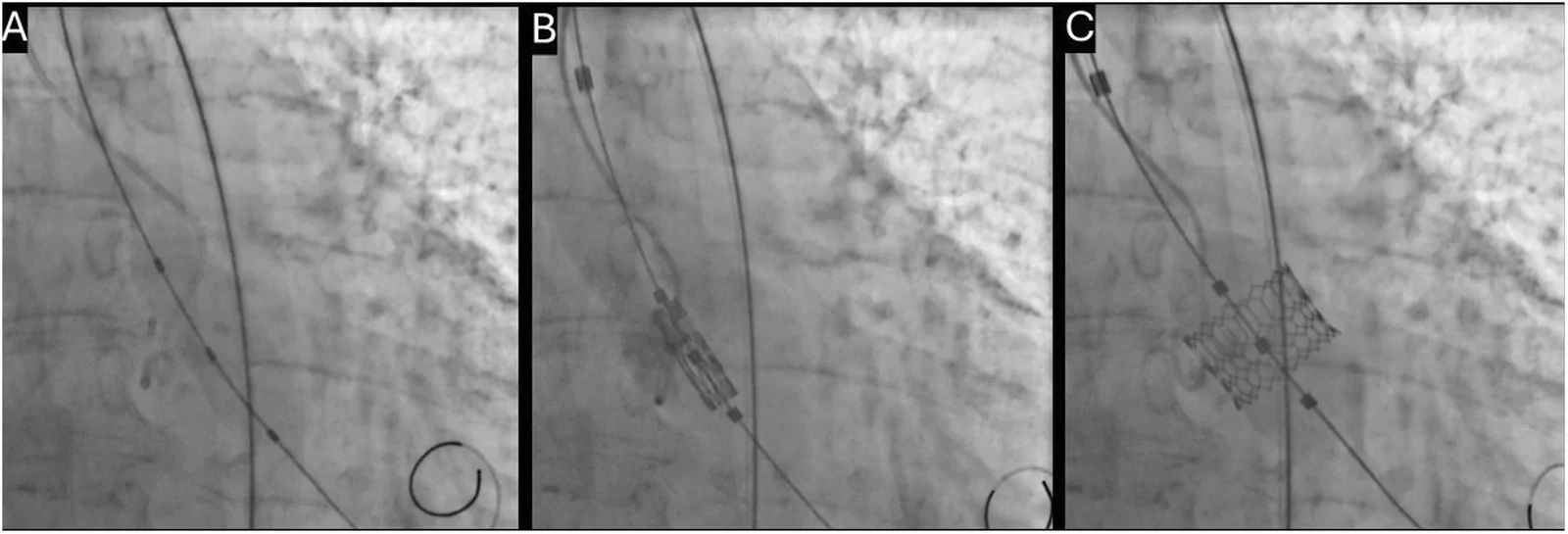
Representative fluoroscopic workflow for ultra–low-contrast myVal TAVI. Representative intraprocedural fluoroscopic still frames illustrating key steps of the ultra–low-contrast implantation workflow. **(A)** Balloon aortic valvuloplasty performed before valve implantation, with the pigtail catheter providing the fluoroscopic aortic-root and annular reference. **(B)** Single limited contrast cine acquisition used to confirm the relationship between the crimped MyVal prosthesis, the pigtail-defined annular plane, and the aortic root before deployment. **(C)** MyVal deployment at maximum balloon expansion, demonstrating prosthesis expansion relative to the annular reference. MyVal, MyVal transcatheter heart valve; TAVI, transcatheter aortic valve implantation.

Valve deployment was then performed under rapid ventricular pacing according to standard balloon-expandable valve technique. Final valve position was assessed fluoroscopically, without routine post-deployment angiography and mainly assessing valve frame expansion. A repeat balloon dilation (double-tap technique) was performed only if frame underexpansion was evident under fluoroscopy. After implantation, procedural success and valve performance were assessed using invasive hemodynamics and transthoracic echocardiography. Hemodynamic assessment included evaluation of residual transvalvular gradient, and separation of aortic and left Ventricular diastolic pressures. Transthoracic echocardiography was used to assess prosthetic valve function, paravalvular regurgitation, left ventricular function, and pericardial effusion. Representative intraprocedural fluoroscopic still frames are shown in Figure 2.

Post-dilatation was performed selectively in cases of suspected relevant residual paravalvular regurgitation, suboptimal valve expansion, or unsatisfactory hemodynamic result. Additional contrast administration beyond the single limited cine acquisition was reserved only for bailout or safety-critical situations, including suspected coronary obstruction, uncertain valve position, significant residual aortic regurgitation, vascular complication, or unexplained hemodynamic instability. Completion of TAVI with total iodinated contrast volume ≤10 mL and without conversion to a standard contrast-guided strategy was considered successful implementation of the ultra–low-contrast protocol.

Total contrast volume was recorded prospectively for every patient and included all contrast administered from vascular access to completion of the procedure. The need for conversion to a standard contrast-guided implantation strategy was recorded as a feasibility failure. Contrast burden was assessed using absolute contrast volume and the contrast volume/eGFR ratio. Contrast volume was analyzed both as a continuous variable and categorically as ≤5 mL, 6–9 mL, and 10 mL. The contrast volume/eGFR ratio was analyzed as a continuous variable and by tertiles. Additional descriptive thresholds included contrast volume/eGFR ratio <0.5 and <1.0.

### 2.6 Transcatheter heart valve platform

All procedures were performed using the MyVal balloon-expandable transcatheter heart valve system. Implanted valve size was selected according to pre-procedural anatomical assessment and Heart Team/procedural operator judgment. MyVal valve size was recorded for each patient and analyzed both as individual valve size and according to size category. Intermediate MyVal sizes were predefined as 21.5 mm, 24.5 mm, 27.5 mm, and 30.5 mm. Standard MyVal sizes were predefined as 20 mm, 23 mm, 26 mm, and 29 mm.

Procedural variables included pre-dilatation, post-dilatation, planned coronary protection, second valve implantation, conversion to surgery, conversion to standard contrast-guided TAVI, procedure time, fluoroscopy time, dose-area product, and length of hospital stay.

### 2.7 Study endpoints

The primary endpoint was procedural feasibility, defined as successful completion of transfemoral MyVal TAVI with total intraprocedural iodinated contrast volume ≤10 mL and without conversion to a standard contrast-guided implantation strategy.

A prespecified renal safety outcome was the occurrence of acute kidney injury after TAVI, assessed during hospitalization and through 30-day follow-up and staged according to KDIGO criteria (13). Because the study was designed primarily to evaluate feasibility and was not powered to detect differences in AKI incidence, renal outcomes were interpreted descriptively.

Secondary endpoints included technical success, device success, early clinical outcomes, procedural complications, echocardiographic valve performance, paravalvular regurgitation, functional status, renal-function trajectory, and 30-day clinical outcomes. TAVI-related clinical endpoints, including technical success, device success, mortality, stroke or transient ischemic attack, myocardial infarction, major vascular complications, major or life-threatening bleeding, valve embolization or migration, annular rupture, coronary obstruction, second valve implantation, emergency cardiac surgery, new permanent pacemaker implantation, and heart failure rehospitalization, were defined according to VARC-3 whenever applicable. VARC-3 is the contemporary endpoint framework for transcatheter and surgical aortic valve clinical research.

Technical success was assessed at the end of the procedure. Device success was assessed at 30 days and incorporated survival, correct valve positioning, absence of procedure-related major device or structural complications, and intended valve performance according to VARC-3 principles. The operational definitions and assessment time points for the procedural, clinical, echocardiographic, and renal endpoints reported in the present study are summarized in Supplementary Table S1.

### 2.8 Renal-function assessment

Serum creatinine and eGFR were recorded at baseline, 24–48 h after TAVI, hospital discharge, and 30 days. For the present analysis, baseline renal function was defined as the most recent serum creatinine and eGFR measurement obtained after pre-procedural contrast-enhanced CT angiography and before TAVI, during the pre-admission or admission laboratory assessment. This value was used as the reference for all post-TAVI renal-function comparisons. When laboratory testing was available both before and after CT angiography, post-CT/pre-TAVI values were used for the main analysis to better reflect renal function immediately before valve implantation. No patient required postponement of TAVI because of renal deterioration after CT angiography. Renal-function trajectory was assessed using absolute serum creatinine values, absolute eGFR values, absolute change in serum creatinine from baseline, absolute change in eGFR from baseline, relative eGFR change, and the proportion of patients with stable or improved renal function.

Stable or improved renal function was defined as no decline in eGFR compared with baseline. Relative eGFR decline >10% and relative eGFR increase >10% were also recorded. New renal replacement therapy was defined as initiation of hemodialysis, hemofiltration, or another renal replacement modality in a patient not receiving chronic dialysis before TAVI.

Because the study focused on contrast minimization and renal safety, renal outcomes were analyzed in the overall cohort and in predefined renal-function subgroups according to baseline eGFR ≥30 versus <30 mL/min/1.73 m^2^. Additional exploratory analyses assessed renal outcomes according to contrast volume category and contrast volume/eGFR ratio tertiles.

Clinical follow-up was extended to 6 months and included all-cause mortality, cardiovascular mortality, heart failure hospitalization, and initiation of new renal replacement therapy. Serum creatinine and eGFR were recorded at 6 months when available. Because systematic laboratory testing was not available for all patients at this time point, 6-month renal-function analyses were performed as available-case analyses, with denominators reported explicitly.

### 2.9 Peri-procedural renal-protection measures

Peri-procedural renal-risk mitigation was performed according to institutional practice and individualized clinical assessment. All patients underwent assessment of volume status, renal function, and heart-failure status before TAVI. Isotonic intravenous hydration was used when clinically appropriate, with the rate and duration individualized according to baseline eGFR, left ventricular function, congestion, and risk of volume overload. In patients with advanced CKD or heart failure, hydration was reduced or avoided when clinically indicated. Low- or iso-osmolar non-ionic iodinated contrast was used for CT angiography and intraprocedural aortography. Potentially nephrotoxic medications, including non-steroidal anti-inflammatory drugs and other avoidable nephrotoxins, were withheld when possible. Diuretics and renin–angiotensin–aldosterone system inhibitors were managed individually according to blood pressure, congestion, renal function, and treating-physician judgment. Metformin, when prescribed, was withheld according to institutional practice in patients with reduced eGFR or other risk factors for contrast-associated AKI and restarted after reassessment of renal function.

### 2.10 Echocardiographic assessment and valve-performance analysis

Transthoracic echocardiography was performed at baseline, before hospital discharge, and at 30-day follow-up. Echocardiographic variables included mean transvalvular gradient, peak aortic velocity, aortic valve area, indexed aortic valve area, left ventricular ejection fraction, and paravalvular regurgitation grade.

Paravalvular regurgitation was graded as none/trace, mild, moderate, or severe using an integrative echocardiographic approach. For analysis, moderate and severe paravalvular regurgitation were considered clinically significant. Residual transvalvular gradient was assessed as a continuous variable and categorically using thresholds of ≥10 mmHg and ≥20 mmHg.

Hemodynamic outcomes were analyzed in the overall cohort and according to annular area and MyVal valve size category. For exploratory annular-size analysis, patients were stratified by annular area <430 mm^2^ versus ≥430 mm^2^. Valve-performance analyses compared standard MyVal sizes with intermediate MyVal sizes.

No external core laboratory was used. Baseline, discharge, and 30-day transthoracic echocardiographic studies were reviewed by an experienced institutional echocardiographer who was not involved in the TAVI procedures and was blinded to intraprocedural contrast volume and clinical outcomes. Prosthetic-valve hemodynamics and paravalvular regurgitation were adjudicated using an integrative echocardiographic approach.

### 2.11 Functional-status assessment

Functional status was assessed using NYHA functional class at baseline, hospital discharge, and 30 days. NYHA class was analyzed both as a four-level ordinal variable and dichotomized as class I/II versus class III/IV. Improvement in functional status was assessed descriptively by changes in NYHA class distribution over time.

### 2.12 Chronological procedural-performance analysis

To evaluate changes in procedural performance over the study period, patients were divided chronologically into the first 25 and last 25 consecutive cases. The following variables were compared between periods: contrast volume, proportion of procedures performed with ≤5 mL contrast, contrast volume/eGFR ratio, procedure time, fluoroscopy time, dose-area product, technical success, post-dilatation, major vascular complications, major or life-threatening bleeding, acute kidney injury, new permanent pacemaker implantation, and 30-day device success.

To explore whether chronological improvements in procedural metrics persisted after accounting for selected markers of case complexity, parsimonious multivariable linear-regression models were constructed for total contrast volume and procedure time. Chronological case number was analyzed as a continuous variable and scaled per 10-case increase. A *post hoc* exploratory anatomical-complexity indicator was defined as the presence of at least one of the following: bicuspid aortic-valve morphology, horizontal aorta, moderate or severe left ventricular outflow tract calcification, low coronary height, or severe iliofemoral calcification or tortuosity. Low coronary height was defined as left-main or right-coronary height <10 mm.

Total contrast volume was modeled as a function of chronological case number, baseline eGFR, and anatomical complexity. Procedure time was modeled as a function of chronological case number, anatomical complexity, and pre-dilatation. To explore factors associated with renal-function trajectory, absolute change in eGFR from baseline to 30 days was modeled as a function of baseline eGFR, contrast volume/eGFR ratio, and peri-procedural hemoglobin decrease.

Covariates were selected *a priori* on clinical grounds. Automated variable-selection procedures were not used. Given the limited sample size, each model was restricted to a maximum of three covariates. Heteroskedasticity-robust standard errors were used, and the analyses were considered exploratory and hypothesis-generating.

### 2.13 Exploratory analyses

Several exploratory analyses were prespecified to provide additional insight into the relationship between contrast burden, renal reserve, procedural complexity, and early outcomes.

First, renal outcomes were compared according to baseline renal function using the eGFR threshold of 30 mL/min/1.73 m^2^. Second, patients were stratified according to absolute contrast volume category: ≤5 mL, 6–9 mL, and 10 mL. Third, patients were divided into tertiles of contrast volume/eGFR ratio. Fourth, correlations were assessed between 30-day eGFR change and baseline/procedural variables, including baseline eGFR, baseline serum creatinine, baseline mean aortic gradient, absolute contrast volume, contrast volume/eGFR ratio, procedure time, fluoroscopy time, dose-area product, hemoglobin decrease, and length of hospital stay.

Because only a small number of acute kidney injury events was expected, and because the anticipated event rate was insufficient for reliable multivariable modeling, no multivariable regression model for acute kidney injury was planned. Instead, continuous change in renal function was used for exploratory association analyses.

### 2.14 Statistical analysis

Continuous variables were assessed for distribution using visual inspection of histograms and Q–Q plots. Normally distributed continuous variables are presented as mean ± standard deviation, while non-normally distributed variables are presented as median with interquartile range. Categorical variables are presented as counts and percentages.

Comparisons between two independent groups were performed using the Student *t*-test or Mann–Whitney *U* test for continuous variables, according to distribution, and the chi-square test or Fisher exact test for categorical variables, as appropriate. Comparisons across more than two groups were performed using one-way analysis of variance or the Kruskal–Wallis test for continuous variables and chi-square or Fisher exact testing for categorical variables.

Serial renal and echocardiographic variables were analyzed descriptively at baseline, 24–48 h, discharge, and 30 days, as applicable. Available paired 6-month renal-function measurements were analyzed separately. Within-patient changes in continuous variables from baseline to follow-up were assessed using paired *t*-tests or Wilcoxon signed-rank tests according to distribution. Changes in categorical or ordinal variables, including NYHA class and paravalvular regurgitation grade, were assessed using appropriate paired non-parametric methods.

Correlation analyses were performed using Spearman rank correlation coefficients because several renal and procedural variables were non-normally distributed. Correlations were considered exploratory and were not adjusted for multiple comparisons.

No formal sample-size calculation was performed, as this was a prospective single-center feasibility and safety study. The sample size was based on consecutive eligible patients treated during the study period. All statistical analyses were performed using R version 4.6.0 (R Foundation for Statistical Computing, Vienna, Austria) in RStudio. A two-sided *p* value <0.05 was considered statistically significant.

### 2.15 Missing data

All 50 enrolled patients had complete baseline, procedural, discharge, and 30-day clinical and renal-function data. Echocardiographic follow-up at discharge and 30 days was also complete. Therefore, no imputation was performed. Analyses were conducted using the full study cohort. Six-month analyses were performed as available-case analyses without imputation.

## 3 Results

### 3.1 Study population

A total of 50 consecutive patients underwent attempted native-valve transfemoral TAVI using the ultra–low-contrast MyVal protocol and were included in the final analysis. Clinical, procedural, echocardiographic, and renal-function data were complete at discharge and at 30 days in all patients. This study was designed primarily to evaluate procedural feasibility and renal safety and was not powered to detect differences in renal or other clinical outcomes. Accordingly, all subgroup comparisons, *p* values, and exploratory models should be interpreted as hypothesis-generating.

The mean age was 82.4 ± 5.8 years, and 24 patients were female. The cohort had a high prevalence of cardiovascular comorbidity, including hypertension in 44 patients, diabetes mellitus in 20 patients, coronary artery disease in 26 patients, atrial fibrillation in 18 patients, and peripheral artery disease in 8 patients. Baseline renal impairment was frequent: median serum creatinine was 1.56 mg/dL, median eGFR was 38 mL/min/1.73 m^2^, and 45 patients had CKD, including 15 patients with eGFR <30 mL/min/1.73 m^2^. No patient was receiving chronic dialysis before TAVI.

Baseline conduction characteristics are summarized in Table 1. First-degree atrioventricular block was present in 6 of 32 patients with assessable atrioventricular conduction. Right bundle branch block, left bundle branch block, and bifascicular block were present in 6, 4, and 3 patients, respectively. Baseline PR interval among patients with assessable atrioventricular conduction was 184 ± 28 ms, and baseline QRS duration was 108 ± 24 ms.

**Table 1:** Baseline clinical and echocardiographic characteristics.

Variable	Overall cohort (*n* = 50)
Age, years	82.4 ± 5.8
Female sex	24 (48.0)
Body mass index, kg/m^2^	27.0 ± 4.2
EuroSCORE II, %	5.7 [4.1–8.3]
STS-PROM score, %	4.6 [3.2–6.1]
Hypertension	44 (88.0)
Diabetes mellitus	20 (40.0)
Coronary artery disease	26 (52.0)
Previous PCI	15 (30.0)
Previous CABG	4 (8.0)
Atrial fibrillation	18 (36.0)
First-degree atrioventricular block	6/32 (18.8)
Right bundle branch block	6 (12.0)
Left bundle branch block	4 (8.0)
Bifascicular block	3 (6.0)
Baseline PR interval, ms, among patients with assessable atrioventricular conduction	184 ± 28
Baseline QRS duration, ms	108 ± 24
Peripheral artery disease	8 (16.0)
Chronic obstructive pulmonary disease	7 (14.0)
Previous stroke or transient ischemic attack	5 (10.0)
Anemia	20 (40.0)
Serum creatinine, mg/dL	1.56 [1.22–2.04]
eGFR, mL/min/1.73 m^2^	38 [29–48]
eGFR ≥60 mL/min/1.73 m^2^	5 (10.0)
eGFR 45–59 mL/min/1.73 m^2^	10 (20.0)
eGFR 30–44 mL/min/1.73 m^2^	20 (40.0)
eGFR 15–29 mL/min/1.73 m^2^	13 (26.0)
eGFR <15 mL/min/1.73 m^2^, not on dialysis	2 (4.0)
NYHA functional class III or IV	35 (70.0)
Left ventricular ejection fraction, %	55 [47–60]
LVEF <50%	17 (34.0)
Aortic valve area, cm^2^	0.66 ± 0.15
Indexed aortic valve area, cm^2^/m^2^	0.36 ± 0.09
Mean aortic gradient, mmHg	45.2 ± 11.8
Peak aortic velocity, m/s	4.2 ± 0.5
Moderate or greater mitral regurgitation	6 (12.0)
Moderate or greater tricuspid regurgitation	13 (26.0)
Pulmonary artery systolic pressure, mmHg	42 [34–52]

Values are mean ± standard deviation, median [interquartile range], or *n* (%). CABG, coronary artery bypass grafting; eGFR, estimated glomerular filtration rate; LVEF, left ventricular ejection fraction; NYHA, New York Heart Association; PCI, percutaneous coronary intervention; STS-PROM, Society of Thoracic Surgeons Predicted Risk of Mortality.

Most patients were highly symptomatic at baseline. NYHA functional class III or IV was present in 35 patients. Severe aortic stenosis was confirmed in all cases, with a mean aortic valve area of 0.66 ± 0.15 cm^2^, mean transvalvular gradient of 45.2 ± 11.8 mmHg, and peak aortic velocity of 4.2 ± 0.5 m/s. Baseline clinical, electrocardiographic, and echocardiographic characteristics are summarized in Table 1.

Aortic valve anatomy was tricuspid in 46 patients and bicuspid in 4 patients. The mean annular area was 476 ± 78 mm^2^, and the mean annular perimeter was 78.2 ± 6.5 mm. Median aortic valve calcium score was 2,170 Agatston units. Severe iliofemoral calcification or tortuosity was present in 18 patients, but transfemoral TAVI was completed in all cases. Low left-main coronary height was present in 5 patients, while low right-coronary height was present in 3 patients. Planned coronary protection was used in 2 patients, and no case of coronary obstruction occurred. Detailed anatomical, access, and MyVal size characteristics are provided in Supplementary Table S2.

All patients underwent standard contrast-enhanced cardiac CT angiography for pre-procedural planning. The median CT contrast volume was 45 mL [interquartile range, 40–50], and the median interval between CT angiography and TAVI was 18 days [11–28]. These pre-procedural contrast data are reported separately from the intraprocedural contrast volume used to define feasibility of the ultra–low-contrast strategy.

### 3.2 Procedural feasibility and intraprocedural contrast exposure

Ultra–low-contrast MyVal TAVI was completed in all 50 patients. No patient required conversion to standard contrast-guided implantation. The median intraprocedural contrast volume was 8 mL [6–10], and no patient received more than 10 mL of iodinated contrast. Thirteen patients received ≤5 mL of contrast, 22 patients received 6–9 mL, and 15 patients received 10 mL. The median contrast volume/eGFR ratio was 0.20 [0.14–0.30], and all patients had a contrast volume/eGFR ratio <1.0.

Technical success was achieved in all patients. There were no cases of intraprocedural death, annular rupture, coronary obstruction, valve embolization, second valve implantation, conversion to surgery, or pericardial tamponade. Pre-dilatation was performed in 30 patients and post-dilatation in 7 patients. Median procedure time was 62 min, median fluoroscopy time was 14.1 min, and median length of hospital stay was 3 days. Procedural characteristics and contrast burden are presented in Table 2.

**Table 2:** Procedural characteristics and contrast burden.

Variable	Overall cohort (*n* = 50)
Transfemoral TAVI	50 (100.0)
MyVal transcatheter heart valve	50 (100.0)
Technical success	50 (100.0)
Pre-dilatation	30 (60.0)
Post-dilatation	7 (14.0)
Planned coronary protection	2 (4.0)
Second valve implantation	0 (0.0)
Conversion to standard contrast-guided TAVI	0 (0.0)
Conversion to surgery	0 (0.0)
Annular rupture	0 (0.0)
Coronary obstruction	0 (0.0)
Valve embolization or migration	0 (0.0)
Pericardial tamponade	0 (0.0)
Contrast volume, mL	8 [6–10]
Mean contrast volume, mL	7.8 ± 2.3
Maximum contrast volume, mL	10
Contrast volume ≤5 mL	13 (26.0)
Contrast volume 6–9 mL	22 (44.0)
Contrast volume 10 mL	15 (30.0)
Contrast volume/eGFR ratio	0.20 [0.14–0.30]
Contrast volume/eGFR ratio <0.5	42 (84.0)
Contrast volume/eGFR ratio <1.0	50 (100.0)
Procedure time, min	62 [52–75]
Fluoroscopy time, min	14.1 [11.0–18.6]
Dose-area product, Gy·cm^2^	42 [29–61]
Length of hospital stay, days	3 [2–5]

Values are mean ± standard deviation, median [interquartile range], or *n* (%). eGFR, estimated glomerular filtration rate; TAVI, transcatheter aortic valve implantation.

### 3.3 Renal outcomes

**Figure 3:**
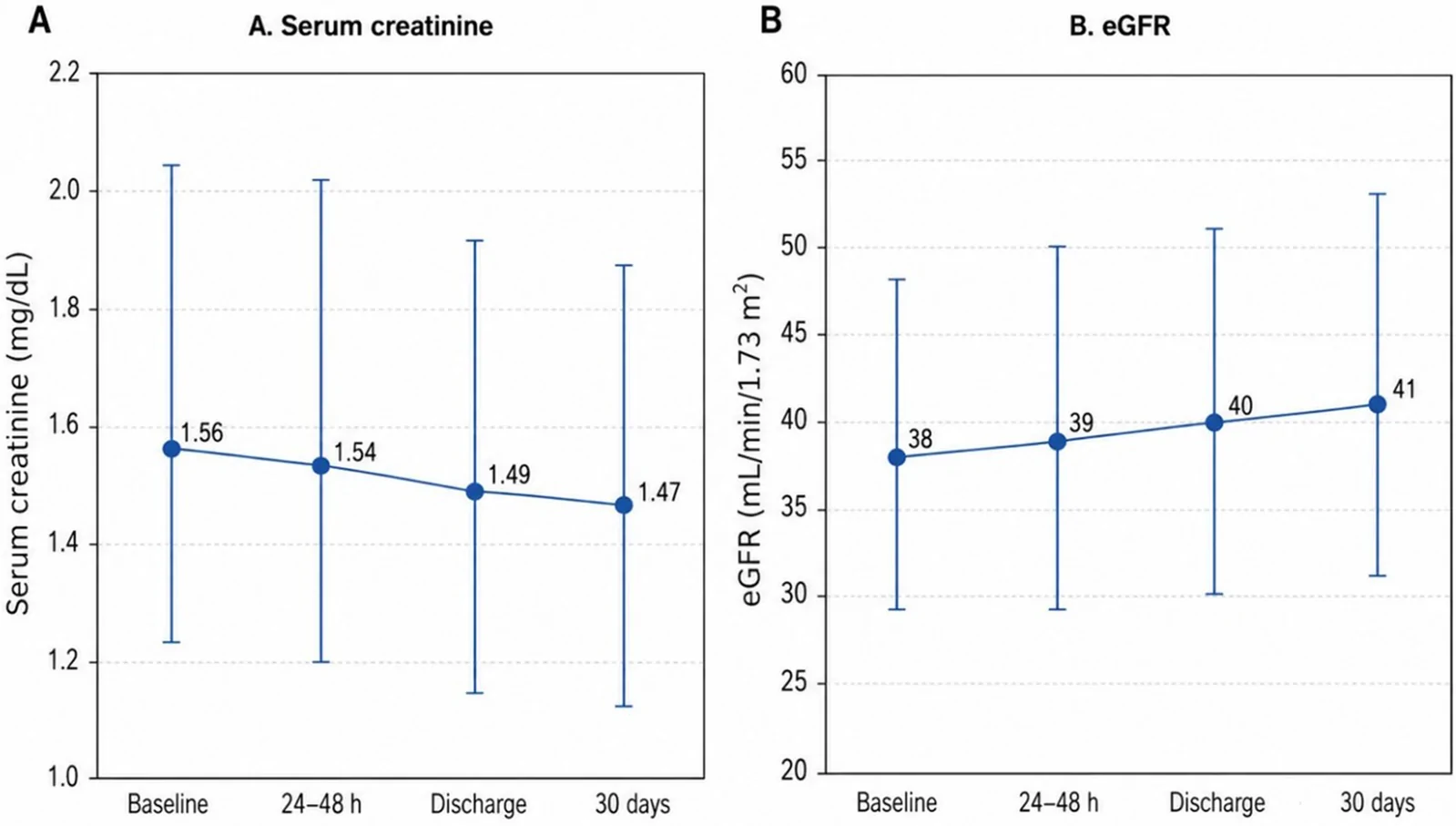
Serial renal-function trajectory after ultra–low-contrast MyVal TAVI. Serial changes in renal function from baseline to 24–48 h, hospital discharge, and 30-day follow-up after ultra–low-contrast transfemoral TAVI using the MyVal transcatheter heart valve. **(A)** Serum creatinine showed no post-procedural increase, with median values of 1.56 mg/dL at baseline, 1.54 mg/dL at 24–48 h, 1.49 mg/dL at discharge, and 1.47 mg/dL at 30 days. **(B)** Estimated glomerular filtration rate remained stable, with median values of 38, 39, 40, and 41 mL/min/1.73 m^2^ at the corresponding time points. Data are presented as median with interquartile range. eGFR, estimated glomerular filtration rate; TAVI, transcatheter aortic valve implantation.

Renal function remained stable after ultra–low-contrast MyVal TAVI. Median serum creatinine was 1.56 mg/dL at baseline, 1.54 mg/dL at 24–48 h, 1.49 mg/dL at discharge, and 1.47 mg/dL at 30 days, with no significant increase from baseline at any post-procedural time point. Median eGFR was 38 mL/min/1.73 m^2^ at baseline, 39 mL/min/1.73 m^2^ at 24–48 h, 40 mL/min/1.73 m^2^ at discharge, and 41 mL/min/1.73 m^2^ at 30 days. Compared with baseline, the median absolute change in serum creatinine was −0.05 mg/dL at discharge and −0.06 mg/dL at 30 days, while the median absolute change in eGFR at 30 days was +3 mL/min/1.73 m^2^. Serial renal-function parameters are shown in Table 3 and illustrated in Figure 3.

**Table 3:** Serial renal-function parameters.

Variable	Baseline	24–48 h	Discharge	30 days	*p* value: baseline vs. 24–48 h	*p* value: baseline vs. discharge	*p* value: baseline vs. 30 days
Serum creatinine, mg/dL	1.56 [1.22–2.04]	1.54 [1.20–2.02]	1.49 [1.14–1.91]	1.47 [1.12–1.87]	0.42	0.11	0.06
eGFR, mL/min/1.73 m^2^	38 [29–48]	39 [29–50]	40 [30–51]	41 [31–53]	0.31	0.08	0.04
Absolute change in creatinine from baseline, mg/dL	—	−0.02 [−0.12 to 0.10]	−0.05 [−0.18 to 0.09]	−0.06 [−0.23 to 0.08]	—	—	—
Absolute change in eGFR from baseline, mL/min/1.73 m^2^	—	+1 [−2 to +4]	+2 [−2 to +5]	+3 [−1 to +6]	—	—	—
Relative eGFR increase >10%	—	11 (22.0)	14 (28.0)	18 (36.0)	—	—	—
Relative eGFR decline >10%	—	7 (14.0)	6 (12.0)	4 (8.0)	—	—	—
Stable or improved renal function	—	43 (86.0)	44 (88.0)	46 (92.0)	—	—	—
Acute kidney injury, any stage	—	1 (2.0)	1 (2.0)	1 (2.0)	—	—	—
Acute kidney injury stage 1	—	1 (2.0)	1 (2.0)	1 (2.0)	—	—	—
Acute kidney injury stage 2 or 3	—	0 (0.0)	0 (0.0)	0 (0.0)	—	—	—
New renal replacement therapy	—	0 (0.0)	0 (0.0)	0 (0.0)	—	—	—

AKI occurred in 1 patient and was limited to KDIGO stage 1. No patient developed stage 2 or stage 3 AKI, and no patient required new renal replacement therapy during the first 30 days. At 30 days, renal function was stable or improved in 46 patients. A relative eGFR decline >10% was observed in 4 patients, whereas a relative eGFR increase >10% was observed in 18 patients.

Values are mean ± standard deviation, median [interquartile range], or *n* (%). *P* values compare paired baseline and follow-up measurements using paired *t*-tests or Wilcoxon signed-rank tests according to distribution.

Patients with baseline eGFR <30 mL/min/1.73 m^2^ had lower renal reserve and higher baseline serum creatinine, but contrast volume remained low in this subgroup. Technical success was achieved in all patients irrespective of baseline renal function. AKI occurred in 1 patient with baseline eGFR ≥30 mL/min/1.73 m^2^ and in no patient with eGFR <30 mL/min/1.73 m^2^. No patient in either subgroup developed stage 2 or stage 3 AKI or required new renal replacement therapy. At 30 days, renal function was stable or improved in 31 of 35 patients with eGFR ≥30 mL/min/1.73 m^2^ and in all 15 patients with eGFR <30 mL/min/1.73 m^2^. Given the very low event count, these subgroup findings are descriptive and should not be interpreted as evidence of differential renal risk or protection. Renal outcomes according to baseline renal function are shown in Supplementary Table S3.

Patients were also stratified according to observed contrast volume. Baseline renal function was numerically worse among patients receiving ≤5 mL contrast, reflecting a more conservative contrast strategy in those with lower renal reserve. No clear numerical differences in 30-day renal outcomes were observed across contrast-volume categories. No patient receiving ≤5 mL contrast developed AKI, required dialysis, or experienced moderate or severe paravalvular regurgitation. Given the low event counts, these exploratory comparisons should be interpreted descriptively. These findings are shown in Supplementary Table S4.

The median contrast volume/eGFR ratio was 0.20. No patient exceeded a contrast volume/eGFR ratio of 1.0, and 42 patients had a ratio <0.5. Across contrast volume/eGFR ratio tertiles, AKI was infrequent and is reported descriptively. No clear numerical differences were observed in absolute eGFR change at 30 days or in the proportion of patients with stable or improved renal function. Renal outcomes according to contrast volume/eGFR ratio tertiles are shown in Supplementary Table S5.

In exploratory correlation analyses, neither absolute contrast volume nor the contrast volume/eGFR ratio was significantly associated with absolute eGFR change at 30 days. Because only 1 AKI event occurred, no regression analysis for AKI was performed. Exploratory correlation analyses focused on continuous renal-function change and are provided in Table 4.

**Table 4:** Exploratory correlations with 30-day eGFR change.

Variable	Correlation with absolute eGFR change at 30 days	*p* value
Baseline eGFR	−0.36	0.010
Baseline serum creatinine	+0.31	0.029
Baseline mean aortic gradient	+0.18	0.21
Contrast volume	−0.06	0.68
Contrast volume/eGFR ratio	+0.09	0.53
Procedure time	−0.14	0.34
Fluoroscopy time	−0.11	0.45
Dose-area product	−0.08	0.58
Hemoglobin decrease	−0.29	0.04
Length of hospital stay	−0.21	0.14

Correlation coefficients are Spearman rank correlation coefficients. eGFR, estimated glomerular filtration rate.

Clinical follow-up at 6 months was available for 49 of 50 patients, while paired renal-function testing was available for 46 patients. Among patients with available paired laboratory measurements, median serum creatinine decreased from 1.56 mg/dL [1.22–2.04] at baseline to 1.39 mg/dL [1.08–1.80] at 6 months (*p* = 0.008), while median eGFR increased from 38 mL/min/1.73 m^2^ [29–48] to 44 mL/min/1.73 m^2^ [34–56] (*p* = 0.003). The median absolute change in eGFR from baseline to 6 months was +5 mL/min/1.73 m^2^ [0 to +9]. Renal function was stable or improved in 41 of 46 patients with available 6-month laboratory follow-up. A relative eGFR increase >10% was observed in 24 patients, whereas a relative eGFR decline >10% occurred in 4 patients. No patient initiated new renal replacement therapy during follow-up. At 6 months, heart failure hospitalization had occurred in 1 of 49 patients, and 1 patient had died from a non-cardiovascular cause. Six-month renal and clinical outcomes are summarized in Table 5.

**Table 5:** Six-month renal and clinical outcomes.

Outcome	Baseline	6 months	*p* value
Serum creatinine, mg/dL	1.56 [1.22–2.04]	1.39 [1.08–1.80]	0.008
eGFR, mL/min/1.73 m^2^	38 [29–48]	44 [34–56]	0.003
Absolute change in serum creatinine from baseline, mg/dL	—	−0.14 [−0.31 to +0.04]	—
Absolute change in eGFR from baseline, mL/min/1.73 m^2^	—	+5 [0 to +9]	—
Relative eGFR increase >10%	—	24/46 (52.2)	—
Relative eGFR decline >10%	—	4/46 (8.7)	—
Stable or improved renal function	—	41/46 (89.1)	—
New renal replacement therapy	0/50 (0.0)	0/49 (0.0)	—
All-cause mortality	—	1/49 (2.0)	—
Cardiovascular mortality	—	0/49 (0.0)	—
Non-cardiovascular mortality	—	1/49 (2.0)	—
Heart-failure hospitalization	—	1/49 (2.0)	—

Values are presented as median [interquartile range] or *n*/*N* (%), as appropriate. *P* values compare paired baseline and 6-month renal-function measurements using the Wilcoxon signed-rank test. eGFR, estimated glomerular filtration rate.

### 3.4 Valve performance and early clinical outcomes

Valve performance and early clinical outcomes were favorable. Mean transvalvular gradient decreased from 45.2 ± 11.8 mmHg at baseline to 8.4 ± 3.0 mmHg at discharge (*p* < 0.001) and 8.7 ± 3.2 mmHg at 30 days (*p* < 0.001). Aortic valve area increased from 0.66 ± 0.15 cm^2^ at baseline to 1.86 ± 0.34 cm^2^ at discharge (*p* < 0.001) and 1.83 ± 0.32 cm^2^ at 30 days (*p* < 0.001). Improvements from baseline were statistically significant for mean transvalvular gradient, peak aortic velocity, aortic valve area, and indexed aortic valve area at both discharge and 30 days, while left ventricular ejection fraction remained stable.

At discharge, paravalvular regurgitation was absent or trace in 34 patients and mild in 16 patients. At 30 days, paravalvular regurgitation was absent or trace in 35 patients and mild in 15 patients. No patient had moderate or severe paravalvular regurgitation at either time point, and no patient had residual mean gradient ≥20 mmHg. Echocardiographic outcomes are presented in Table 6.

**Table 6:** Echocardiographic outcomes.

Variable	Baseline	Discharge	30 days	*p* value: baseline vs. discharge	*p* value: baseline vs. 30 days
Mean aortic gradient, mmHg	45.2 ± 11.8	8.4 ± 3.0	8.7 ± 3.2	<0.001	<0.001
Peak aortic velocity, m/s	4.2 ± 0.5	1.9 ± 0.3	1.9 ± 0.3	<0.001	<0.001
Aortic valve area, cm^2^	0.66 ± 0.15	1.86 ± 0.34	1.83 ± 0.32	<0.001	<0.001
Indexed aortic valve area, cm^2^/m^2^	0.36 ± 0.09	1.01 ± 0.20	0.99 ± 0.19	<0.001	<0.001
Left ventricular ejection fraction, %	55 [47–60]	56 [48–61]	57 [49–62]	0.18	0.07
Residual mean gradient ≥10 mmHg	—	10 (20.0)	11 (22.0)	—	—
Residual mean gradient ≥20 mmHg	—	0 (0.0)	0 (0.0)	—	—
No/trace paravalvular regurgitation	—	34 (68.0)	35 (70.0)	—	—
Mild paravalvular regurgitation	—	16 (32.0)	15 (30.0)	—	—
Moderate paravalvular regurgitation	—	0 (0.0)	0 (0.0)	—	—
Severe paravalvular regurgitation	—	0 (0.0)	0 (0.0)	—	—

Values are mean ± standard deviation, median [interquartile range], or *n* (%). *P* values compare paired baseline and follow-up measurements using paired *t*-tests or Wilcoxon signed-rank tests according to distribution.

Values are mean ± standard deviation, median [interquartile range], or *n* (%). *P* values compare paired baseline and follow-up measurements using paired *t*-tests or Wilcoxon signed-rank tests according to distribution.

There were no in-hospital or 30-day deaths. No stroke, transient ischemic attack, myocardial infarction, coronary obstruction, annular rupture, valve embolization, second valve implantation, or emergency cardiac surgery occurred. One patient developed a major vascular complication requiring endovascular treatment, three additional patients had minor access-site complications, one patient experienced major bleeding, and two patients required red blood cell transfusion. One patient was rehospitalized for heart failure within 30 days. New permanent pacemaker implantation was required in 4 patients. In-hospital and 30-day clinical outcomes are summarized in Table 7.

**Table 7:** In-hospital and 30-day clinical outcomes.

Outcome	In-hospital/discharge	30 days
Technical success	50 (100.0)	—
Device success	—	50 (100.0)
All-cause death	0 (0.0)	0 (0.0)
Cardiovascular death	0 (0.0)	0 (0.0)
Stroke or transient ischemic attack	0 (0.0)	0 (0.0)
Myocardial infarction	0 (0.0)	0 (0.0)
Coronary obstruction	0 (0.0)	0 (0.0)
Annular rupture	0 (0.0)	—
Valve embolization or migration	0 (0.0)	0 (0.0)
Second valve implantation	0 (0.0)	—
Emergency cardiac surgery	0 (0.0)	—
Major vascular complication	1 (2.0)	1 (2.0)
Minor vascular/access-site complication	3 (6.0)	3 (6.0)
Major or life-threatening bleeding	1 (2.0)	1 (2.0)
Red blood cell transfusion	2 (4.0)	2 (4.0)
Acute kidney injury, any stage	1 (2.0)	1 (2.0)
Acute kidney injury stage 2 or 3	0 (0.0)	0 (0.0)
New renal replacement therapy	0 (0.0)	0 (0.0)
New permanent pacemaker implantation	4 (8.0)	4 (8.0)
Heart failure rehospitalization	—	1 (2.0)
NYHA functional class I or II	39 (78.0)	45 (90.0)

Values are *n* (%). NYHA, New York Heart Association.

Functional status improved after TAVI. At baseline, 35 patients were in NYHA functional class III or IV. At discharge, 39 patients were in NYHA functional class I or II, increasing to 45 patients at 30 days. The proportion of patients in NYHA functional class III or IV decreased from 70.0% at baseline to 22.0% at discharge and 10.0% at 30 days.

Intermediate MyVal sizes were used in 22 patients. Patients treated with intermediate-size valves had similar early valve performance compared with those treated with standard-size valves, including comparable 30-day mean gradient, aortic valve area, and paravalvular regurgitation. New permanent pacemaker implantation rates were also numerically similar between groups. These exploratory findings are shown in Supplementary Table S6.

Patients with smaller annular dimensions had numerically higher residual gradients, although valve performance remained favorable across the annular-size spectrum. Patients with annular area <430 mm^2^ had higher mean gradients at discharge and 30 days compared with those with annular area ≥430 mm^2^. However, no patient in either group had residual mean gradient ≥20 mmHg or moderate or severe paravalvular regurgitation. Hemodynamic outcomes according to annular area are shown in Supplementary Table S7.

### 3.5 Exploratory procedural-performance analyses

When the cohort was divided chronologically, contrast use decreased over time. Median contrast volume was 9 mL in the first 25 patients and 6 mL in the last 25 patients. The proportion of procedures performed with ≤5 mL contrast increased from 4.0% to 48.0%. Procedure time, fluoroscopy time, and dose-area product were also lower in the later experience, while technical success remained 100% in both periods. Adverse-event counts were low in both chronological periods and did not increase numerically in the later experience. The chronological analysis is shown in Supplementary Table S8.

In exploratory multivariable analyses, later chronological case sequence remained independently associated with lower contrast use after adjustment for baseline eGFR and anatomical complexity. Each 10-case increase was associated with a 0.74-mL reduction in total contrast volume [adjusted β, −0.74 mL; 95% confidence interval (CI), −1.08 to −0.40; *p* < 0.001]. Anatomical complexity was associated with a modest increase in contrast volume (adjusted β, +0.83 mL; 95% CI, +0.12 to +1.54; *p* = 0.023), whereas baseline eGFR was not significantly associated with contrast use.

Later chronological case sequence was also associated with shorter procedure duration after adjustment for anatomical complexity and pre-dilatation. Each 10-case increase was associated with a 4.6-minute reduction in procedure time (adjusted β, −4.6 min; 95% CI, −7.3 to −1.9; *p* = 0.001). Anatomical complexity and pre-dilatation were associated with longer procedures.

In the renal-function model, lower baseline eGFR was associated with a greater increase in eGFR at 30 days (adjusted β per 10-mL/min/1.73 m^2^ increase in baseline eGFR, −1.3 mL/min/1.73 m^2^; 95% CI, −2.2 to −0.4; *p* = 0.006). Contrast volume/eGFR ratio was not independently associated with 30-day eGFR change (adjusted β, +0.8 mL/min/1.73 m^2^; 95% CI, −2.6 to +4.2; *p* = 0.64). Greater peri-procedural hemoglobin decrease was associated with less favorable renal-function change (adjusted β per 1-g/dL decrease, −1.1 mL/min/1.73 m^2^; 95% CI, −2.0 to −0.2; *p* = 0.018). These findings are presented in Supplementary Table S9 and should be interpreted as exploratory.

## 4 Discussion

In this prospective single-center study of 50 consecutive patients undergoing transfemoral TAVI exclusively with the MyVal transcatheter heart valve, an intraprocedural ultra–low-contrast strategy was feasible and was completed successfully in all cases. TAVI was performed with a median intraprocedural contrast volume of 8 mL, no patient received more than 10 mL of iodinated contrast, and no case required conversion to a standard contrast-guided implantation strategy. Technical success was achieved in all patients, early valve performance was preserved, and no patient had moderate or severe paravalvular regurgitation at discharge or 30 days. Renal function remained stable in most patients, AKI occurred in one patient and was limited to KDIGO stage 1, and no patient developed stage 2 or 3 AKI or required new renal replacement therapy.

These findings should be interpreted strictly as feasibility and early safety observations. Because the study was single-arm and did not include a contemporaneous standard-contrast comparator group, it cannot demonstrate renal protection, reduction in AKI, or superiority over conventional contrast-guided TAVI. The observed renal trajectory cannot be attributed specifically to intraprocedural contrast minimization and may also reflect baseline renal reserve, peri-procedural management, hemodynamic improvement after valve replacement, regression to the mean, and other unmeasured factors.

### 4.1 Clinical relevance of renal-risk mitigation in contemporary TAVI

AKI remains one of the most clinically relevant non-cardiac complications after TAVI. Although its incidence varies widely according to population risk, endpoint definition, procedural era, and AKI classification system, it has consistently been associated with worse early and late outcomes (4, 6, 14). In a contemporary analysis, AKI occurred in approximately one in six patients after TAVR and was associated with a substantially higher risk of mortality at 1 year (4). In addition, systematic reviews and observational studies have shown that baseline renal dysfunction, diabetes mellitus, bleeding, vascular complications, hemodynamic instability, and contrast exposure contribute to the risk of post-TAVI AKI (4–6, 14).

The pathophysiology of AKI after TAVI is complex and multifactorial. Potential mechanisms include baseline renal vulnerability, transient hypotension, rapid pacing, low cardiac output, bleeding-related renal hypoperfusion, anemia, systemic inflammation, cholesterol embolization, vascular complications, nephrotoxic medications, and iodinated contrast exposure (5, 6, 14). This multifactorial nature is important for interpretation of the present study. Contrast minimization cannot eliminate all causes of AKI after TAVI. However, contrast exposure remains one of the few procedural variables that can be anticipated, measured, and actively reduced. Therefore, strategies aimed at minimizing iodinated contrast are particularly relevant in elderly patients with CKD, in whom renal reserve is limited and even small deteriorations in kidney function may affect recovery, hospitalization, and subsequent prognosis (5, 7–9).

### 4.2 Interpretation of the low AKI rate

AKI occurred in only one patient and was limited to stage 1. Although this descriptive finding is reassuring, the event count is too low to support conclusions regarding AKI risk reduction, subgroup differences, or predictors of renal injury. The observed renal trajectory, with stable or modestly improved median serum creatinine and eGFR from baseline to 30 days, indicates that no apparent early renal penalty was observed in this cohort. However, these findings should be interpreted descriptively and cannot establish a renal-protective effect of the ultra–low-contrast strategy.

However, the interpretation of post-TAVI renal improvement requires caution. Improvement in renal function after successful relief of severe aortic stenosis may reflect improved forward flow, reduced left-sided filling pressures, improved renal perfusion, lower venous congestion, and reversal of cardiorenal physiology (7). Therefore, stable or improved renal function after TAVI may result from both avoidance of procedural renal injury and hemodynamic benefit after valve replacement. Because this study was not randomized and did not include a standard-contrast control group, it cannot determine the relative contribution of contrast minimization versus post-TAVI hemodynamic improvement. The most scientifically accurate conclusion is therefore that ultra–low-contrast MyVal TAVI was associated with preserved renal function and a low incidence of early AKI, rather than that it definitively prevented AKI.

### 4.3 Contrast volume adjusted for renal function

A key aspect of the present analysis is the use of contrast volume/eGFR ratio in addition to absolute contrast volume. Absolute contrast volume alone does not fully capture renal risk, because the same contrast dose may have different implications in a patient with preserved renal function compared with a patient with advanced CKD. Previous studies have shown that renal function–adjusted contrast exposure, including contrast volume-to-eGFR or contrast volume-to-creatinine clearance ratios, is associated with AKI and adverse early outcomes after TAVI (7–9, 15). In particular, recent data demonstrated that a contrast volume/eGFR threshold around 3.3–3.6 was associated with increased AKI, new renal replacement therapy, and 30-day mortality after TAVI (9).

In the present cohort, the median contrast volume/eGFR ratio was 0.20, and all patients remained below 1.0. This is substantially lower than thresholds previously associated with AKI and mortality (7–9, 15). The absence of an association between contrast volume/eGFR ratio and renal deterioration in the present study should therefore not be interpreted as evidence that the ratio is unimportant. Rather, it likely reflects the narrow and uniformly low range of contrast exposure achieved in this cohort. When contrast volume is constrained to ≤10 mL, the biological gradient required to detect a dose-response relationship may no longer be present. This observation supports a practical concept: in high-risk renal patients, contrast exposure should be interpreted relative to baseline renal function, and maintaining a very low contrast volume/eGFR ratio may be a useful procedural target.

### 4.4 Advanced CKD subgroup

Patients with eGFR <30 mL/min/1.73 m^2^ represent a particularly important subgroup because they are at increased risk of renal deterioration, dialysis, and adverse outcomes after invasive cardiovascular procedures. In the present study, ultra–low-contrast MyVal TAVI was feasible in all patients with advanced CKD. No patient in this subgroup developed AKI, required new renal replacement therapy, or died within 30 days. Renal function was stable or improved in all patients with eGFR <30 mL/min/1.73 m^2^ at 30 days.

These findings are encouraging and support the potential utility of ultra–low-contrast TAVI in patients with advanced renal impairment. They are also consistent with the broader rationale of contrast-sparing TAVI strategies, which have been developed specifically to address the high-risk CKD population (10, 16, 17). Nevertheless, the subgroup included only 15 patients, and the study was not powered for definitive subgroup inference. The absence of AKI in this subgroup should therefore be viewed as hypothesis-generating. Larger CKD-focused prospective studies are needed to determine whether ultra–low-contrast TAVI reduces AKI, dialysis requirement, length of stay, or long-term renal decline compared with standard-contrast TAVI.

### 4.5 Six-month renal and clinical trajectory

The extended follow-up provides additional descriptive context regarding the renal and clinical trajectory after ultra–low-contrast MyVal TAVI. Renal function remained broadly stable or improved among patients with available paired laboratory measurements, with a reduction in serum creatinine and an increase in estimated glomerular filtration rate compared with baseline. No patient initiated new renal replacement therapy during follow-up.

The observed improvement in renal function may plausibly reflect, at least in part, the hemodynamic benefit of relieving severe aortic stenosis. Improved forward flow, enhanced renal perfusion, and reduced venous congestion may contribute to partial reversal of cardiorenal dysfunction, particularly in patients with impaired baseline renal reserve. However, this mechanism cannot be established from the present data because cardiac output, filling pressures, and congestion markers were not assessed serially.

Importantly, the longer-term renal findings should not be interpreted as evidence that contrast minimization independently improves renal function. The absence of a contemporaneous standard-contrast comparator group precludes separation of the potential contribution of reduced intraprocedural contrast exposure from the expected hemodynamic effects of successful valve replacement, regression to the mean, medication changes, and other post-procedural factors. In addition, renal-function testing was not available for the entire cohort at follow-up. These findings should therefore be regarded as descriptive and hypothesis-generating.

### 4.6 Feasibility of ultra–low-contrast TAVI compared with non-contrast and zero-contrast strategies

The present study adds to a growing body of evidence supporting contrast-sparing TAVI. A prospective pilot study of non-contrast TAVI in patients with CKD showed that a zero-contrast planning and implantation strategy using non-contrast imaging modalities and self-expanding valves was feasible, with 92% device success and 92% early safety at 30 days (10). More recently, a zero-contrast TAVI registry in patients with severe renal dysfunction demonstrated 100% procedural success and comparable procedural outcomes compared with standard practice, with no moderate or severe paravalvular leak in the zero-contrast group (17). Another contemporary proof-of-concept study described a no-contrast TAVR evaluation and implantation algorithm in CKD patients, reporting 100% VARC-3 technical success, no index-procedure deaths, no valve embolization, and no need for a second valve (16).

The present study differs from these reports in several ways. First, it evaluated an ultra–low-contrast rather than fully zero-contrast strategy. In the present protocol, standard contrast-enhanced CT angiography was used for pre-procedural planning, while intraprocedural contrast was restricted to a single limited cine acquisition using ≤10 mL. This approach may represent a pragmatic compromise between contrast minimization and procedural reassurance, particularly during early implementation of a contrast-minimization program or in cases in which complete contrast avoidance may reduce operator confidence. A limited angiographic confirmation step may be useful in balloon-expandable valve implantation, uncertain fluoroscopic orientation, low coronary heights, heavy calcification, horizontal aorta, challenging aortic-root anatomy, or planned coronary protection.

Second, the present study used exclusively the MyVal balloon-expandable platform, whereas several prior contrast-sparing studies used self-expanding valves or mixed strategies (10, 16, 17). Third, intraprocedural contrast exposure was consistently restricted to ≤10 mL, providing a reproducible operational definition of ultra–low-contrast TAVI. Fourth, renal function was assessed serially through 30 days, and contrast burden was evaluated both absolutely and relative to eGFR. Thus, ultra–low-contrast and zero-contrast TAVI should be viewed as complementary rather than competing strategies. Zero-contrast TAVI may be preferable in patients with extreme renal vulnerability, advanced CKD approaching dialysis, previous severe contrast reaction, or a clinical need to avoid any additional iodinated contrast exposure. However, it requires dedicated non-contrast imaging protocols, substantial operator experience, and high confidence in non-angiographic landmarks. Ultra–low-contrast TAVI may be easier to implement more broadly, but it does not eliminate cumulative iodinated contrast exposure when contrast-enhanced CT is used for planning.

### 4.7 Procedural and valve-performance findings

An important strength of the present study is the exclusive use of the MyVal transcatheter heart valve, which reduces device-related heterogeneity and allows interpretation of the ultra–low-contrast workflow within a single balloon-expandable platform. MyVal has an expanding evidence base in native aortic stenosis: the MyVal-1 first-in-human study demonstrated early safety and efficacy, the LANDMARK randomized trial showed non-inferiority of the MyVal THV series compared with contemporary transcatheter valves at 30 days, subsequent 1-year LANDMARK results reported comparable clinical and hemodynamic outcomes versus contemporary SAPIEN/Evolut platforms, and real-world data have supported favorable MyVal performance through 2 years (11, 12, 18, 19). In this context, the present study extends the MyVal literature to an ultra–low-contrast TAVI workflow.

Although renal outcomes were the primary focus, preservation of valve performance is essential when angiographic confirmation is minimized. In the present cohort, technical success was achieved in all patients, no conversion to standard contrast-guided implantation was required, and early valve performance was preserved, with marked gradient reduction, no residual mean gradient ≥20 mmHg, and no moderate or severe paravalvular regurgitation at discharge or 30 days. Intermediate MyVal sizes were used in 44% of patients and were associated with similar early valve performance compared with standard sizes. Patients with smaller annuli had higher residual gradients, as expected because smaller annuli and prostheses are associated with higher transprosthetic gradients and greater prosthesis–patient mismatch risk (20), but absolute gradients remained low and no patient had clinically relevant residual obstruction. The absence of moderate or severe paravalvular regurgitation is also relevant, because residual regurgitation after TAVI remains associated with adverse outcomes even in the contemporary device era (21). New permanent pacemaker implantation occurred in 8.0% of patients; however, the study was not powered to assess predictors of conduction disturbance, and implantation depth was not prospectively recorded. Overall, these secondary procedural findings suggest that the ultra–low-contrast workflow did not compromise valve positioning, expansion, or early hemodynamic performance, but they should be interpreted as supportive feasibility findings rather than as primary efficacy endpoints.

### 4.8 Chronological performance and learning curve

The chronological analysis showed progressive reductions in contrast volume, contrast volume/eGFR ratio, procedure time, fluoroscopy time, and dose-area product over the study period. The proportion of procedures performed with ≤5 mL of contrast also increased markedly over time. Exploratory adjusted analyses were added to assess whether the reductions in contrast use and procedure duration persisted after accounting for selected markers of case complexity. The associations between later case sequence and improved procedural metrics remained evident after adjustment, suggesting progressive workflow refinement and increasing team familiarity with the ultra–low-contrast protocol.

These findings should be interpreted cautiously. The study was not designed to characterize a formal learning curve, and chronological case number may capture several factors beyond operator experience, including evolving case selection, refined pre-procedural planning, procedural standardization, and improved team coordination. Furthermore, the *post hoc* anatomical-complexity indicator cannot account for all relevant dimensions of procedural difficulty. Residual confounding therefore remains possible.

### 4.9 Clinical implications

The present findings have practical implications primarily for procedural implementation rather than for comparative efficacy. They suggest that, intraprocedural contrast exposure can be restricted to ≤10 mL without compromising immediate procedural success or early valve performance. The study does not establish that this approach reduces AKI compared with conventional TAVI. Rather, it provides a standardized framework that can be evaluated prospectively in comparative studies.

Third, the results support further evaluation of renal-adjusted contrast metrics. The contrast volume/eGFR ratio is simple, clinically intuitive, and supported by prior TAVI literature (7–9, 15). In this study, all patients remained well below previously proposed high-risk contrast volume/eGFR thresholds, which may have contributed to the observed renal trajectory, although this cannot be established without a comparator. Fourth, the findings suggest that contrast minimization should be integrated into a broader renal-risk mitigation strategy, including avoidance of bleeding and hypotension, meticulous vascular access, careful hemodynamic management, individualized hydration, avoidance of nephrotoxins, and systematic post-procedural renal monitoring.

Finally, the exclusive use of the MyVal platform suggests that a balloon-expandable valve can be used successfully within an ultra–low-contrast workflow. This is important because many prior non-contrast TAVI experiences have focused on self-expanding valves. The present study therefore broadens the procedural applicability of contrast-sparing TAVI strategies.

### 4.10 Study limitations

Several limitations should be acknowledged. First, this was a single-center study with a relatively small sample size. Although the prospective design and consecutive enrollment strengthen internal validity, the findings may not be generalizable to centers with different experience, procedural workflows, imaging protocols, or valve-platform preferences. Second, the study was single-arm and did not include a contemporaneous standard-contrast comparator group. Therefore, the observed low AKI rate and stable renal-function trajectory cannot be attributed causally to contrast minimization and should not be interpreted as evidence of superiority over conventional contrast-guided TAVI. A historical control group was not introduced solely for the purposes of comparison because differences in procedural era, operator experience, case selection, and peri-procedural management could generate substantial confounding. Third, the study should not be interpreted as an all-comers TAVI screening registry. It represents a prospective feasibility cohort of consecutive patients in whom native-valve transfemoral MyVal TAVI was attempted using an ultra–low-contrast protocol. Although no specific anatomical-complexity feature was considered an automatic exclusion criterion after selection of transfemoral MyVal TAVI, the findings should not be extrapolated automatically to patients requiring non-transfemoral access, another valve platform, emergency TAVI, or serial intraprocedural angiographic assessment. Fourth, the number of AKI events was very low, precluding meaningful multivariable modeling of AKI predictors. For this reason, the exploratory analyses focused on continuous renal-function change rather than categorical AKI prediction. The absence of significant associations between contrast metrics and renal outcomes should therefore be interpreted cautiously. Fifth, although clinical follow-up was extended, renal-function testing was not available for the entire cohort at the longer-term time point. The renal analysis was therefore performed as an available-case analysis and may be affected by ascertainment bias. Furthermore, the follow-up duration remains insufficient to evaluate long-term renal decline, recurrent heart-failure hospitalization, late mortality, valve thrombosis, or structural valve deterioration. Sixth, renal function was assessed using serum creatinine and eGFR, which may be influenced by hydration status, muscle mass, frailty, peri-procedural hemodynamics, and laboratory variability. More sensitive biomarkers of tubular injury were not available. In addition, urinary protein excretion, albuminuria, CKD etiology, and systematic nephrology follow-up were not prospectively collected. These factors may influence renal trajectory independently and may have resulted in residual confounding. Therefore, the renal analyses should be interpreted as descriptive assessments of creatinine-based kidney-function trajectory rather than as comprehensive characterization of CKD progression. Also, the evaluated protocol focused on intraprocedural contrast minimization and should not be interpreted as a fully contrast-free patient pathway. Standard contrast-enhanced cardiac CT angiography was used for pre-procedural planning. Although the CT contrast volume and CT-to-TAVI interval are now reported explicitly, cumulative iodinated contrast exposure may have influenced renal outcomes and should be considered when interpreting the observed renal-function trajectory. Finally, the study evaluated only the MyVal platform; therefore, the findings should not be extrapolated automatically to other balloon-expandable or self-expanding transcatheter heart valves.

## 5 Conclusion

In this prospective single-center feasibility study, an intraprocedural ultra–low-contrast transfemoral TAVI strategy using exclusively the MyVal transcatheter heart valve was successfully implemented in the studied cohort, with no need for conversion to standard contrast-guided implantation. Early procedural success and valve performance were preserved, and renal function remained stable or improved during follow-up among patients with available laboratory data. These descriptive findings do not establish renal protection, AKI reduction, or superiority over standard contrast-guided TAVI. Larger multicenter comparative studies with standardized cumulative contrast assessment, systematic renal-function monitoring, and longer follow-up are required to determine whether this strategy improves renal or clinical outcomes.

## Data Availability

The raw data supporting the conclusions of this article will be made available by the authors, without undue reservation.
