# Supplementary material for: Ultra–low-contrast transfemoral TAVI using the MyVal transcatheter heart valve: a prospective single-center feasibility and safety study
Source: Front Cardiovasc Med. 2026 Aug 26;13:1878620. doi: 10.3389/fcvm.2026.1878620 (PMC13553938; doi:10.3389/fcvm.2026.1878620)
Supplement: Supplementary file 1 [file Table1.docx]

Supplementary Material

# 1 Supplementary Data

**Supplementary Table S1. Operational definitions and assessment time points for study endpoints**

| Endpoint | Operational definition used in the present study | Assessment time point |
| --- | --- | --- |
| Procedural feasibility | Completion of transfemoral MyVal TAVI with total intraprocedural iodinated contrast volume ≤10 mL and without conversion to a standard contrast-guided implantation strategy | End of procedure |
| Conversion to standard contrast-guided TAVI | Requirement for additional angiographic acquisitions beyond the prespecified ultra–low-contrast workflow because of a safety-critical or bailout indication | Intraprocedural |
| Technical success | Successful vascular access, delivery and deployment of a single prosthetic valve in the intended position, successful retrieval of the delivery system, freedom from intraprocedural mortality, and freedom from surgery or intervention related to a device-related, major vascular/access-related, or cardiac structural complication | Exit from procedure room |
| Device success | Technical success, survival, correct positioning of a single prosthetic valve, absence of device-related surgery or intervention, and intended prosthetic-valve performance | 30 days |
| Intended prosthetic-valve performance | Mean transvalvular gradient <20 mmHg, absence of moderate or severe paravalvular regurgitation, and absence of clinically relevant prosthetic-valve dysfunction | Discharge and 30 days |
| All-cause mortality | Death from any cause | In-hospital, 30 days, and 6 months |
| Cardiovascular mortality | Death attributable to a cardiovascular cause or death of uncertain cause unless clearly non-cardiovascular | 6 months |
| Stroke | Acute neurological dysfunction attributable to a focal or global vascular brain, spinal-cord, or retinal injury | In-hospital and 30 days |
| Transient ischemic attack | Transient focal neurological symptoms without evidence of acute infarction | In-hospital and 30 days |
| Myocardial infarction | Periprocedural or spontaneous myocardial infarction adjudicated according to VARC-3 principles | In-hospital and 30 days |
| Major vascular complication | Major vascular, access-related, or access-site complication adjudicated according to VARC-3 principles | In-hospital and 30 days |
| Minor vascular/access-site complication | Vascular or access-site complication not meeting criteria for a major event | In-hospital and 30 days |
| Major or life-threatening bleeding | Clinically relevant bleeding adjudicated according to VARC-3 principles | In-hospital and 30 days |
| Acute kidney injury | Increase in serum creatinine meeting KDIGO criteria; staged as stage 1, 2, or 3 | During hospitalization and through 30 days |
| New renal replacement therapy | Initiation of hemodialysis, hemofiltration, or another renal-replacement modality in a patient not receiving chronic dialysis before TAVI | In-hospital, 30 days, and 6 months |
| New permanent pacemaker implantation | Permanent pacemaker implanted after TAVI because of a new or worsened conduction disturbance | In-hospital and 30 days |
| Heart-failure rehospitalization | Unplanned hospitalization for signs or symptoms of heart failure requiring inpatient management | 30 days and 6 months |
| Paravalvular regurgitation | Echocardiographic grading as none/trace, mild, moderate, or severe using an integrative approach | Discharge and 30 days |
| Stable or improved renal function | No decline in eGFR compared with baseline | 30 days and 6 months |
| Relative eGFR decline >10% | Reduction in eGFR of >10% compared with baseline | 30 days and 6 months |
| Relative eGFR increase >10% | Increase in eGFR of >10% compared with baseline | 30 days and 6 months |

Definitions were aligned with Valve Academic Research Consortium-3 criteria whenever applicable. Acute kidney injury was staged according to Kidney Disease: Improving Global Outcomes criteria. eGFR, estimated glomerular filtration rate; KDIGO, Kidney Disease: Improving Global Outcomes; TAVI, transcatheter aortic valve implantation; VARC-3, Valve Academic Research Consortium-3.

**Supplementary Table S2. Anatomical/access characteristics and MyVal size distribution**

| **Variable** | **Overall cohort (n = 50)** |
| --- | --- |
| Tricuspid aortic valve | 46 (92.0) |
| Bicuspid aortic valve | 4 (8.0) |
| Annular area, mm² | 476 ± 78 |
| Annular perimeter, mm | 78.2 ± 6.5 |
| Annular eccentricity index | 0.22 ± 0.08 |
| Aortic valve calcium score, Agatston units | 2,170 [1,440–3,260] |
| Moderate/severe LVOT calcification | 8 (16.0) |
| Sinus of Valsalva diameter, mm | 31.0 ± 3.3 |
| Sinotubular junction diameter, mm | 28.1 ± 3.1 |
| Left main coronary height, mm | 13.2 ± 3.0 |
| Right coronary height, mm | 15.4 ± 3.4 |
| Low left main coronary height | 5 (10.0) |
| Low right coronary height | 3 (6.0) |
| Horizontal aorta | 14 (28.0) |
| Minimal iliofemoral luminal diameter, mm | 6.4 ± 0.9 |
| Severe iliofemoral calcification | 15 (30.0) |
| Severe iliofemoral tortuosity | 11 (22.0) |
| **MyVal valve size** |  |
| 20 mm | 3 (6.0) |
| 21.5 mm | 5 (10.0) |
| 23 mm | 10 (20.0) |
| 24.5 mm | 8 (16.0) |
| 26 mm | 10 (20.0) |
| 27.5 mm | 6 (12.0) |
| 29 mm | 5 (10.0) |
| 30.5 mm | 3 (6.0) |
| Intermediate MyVal sizes | 22 (44.0) |

Values are mean ± standard deviation, median [interquartile range], or n (%). LVOT, left ventricular outflow tract.

**Supplementary Table S3. Outcomes according to baseline renal function**

| **Variable** | **eGFR ≥30 mL/min/1.73 m² (n = 35)** | **eGFR <30 mL/min/1.73 m² (n = 15)** | **p value** |
| --- | --- | --- | --- |
| Age, years | 82.0 ± 5.7 | 83.2 ± 6.1 | 0.50 |
| Female sex | 16 (45.7) | 8 (53.3) | 0.62 |
| EuroSCORE II, % | 5.2 [3.8–7.4] | 7.1 [5.1–9.6] | 0.04 |
| Baseline creatinine, mg/dL | 1.35 [1.10–1.62] | 2.28 [1.94–2.86] | <0.001 |
| Baseline eGFR, mL/min/1.73 m² | 43 [36–52] | 24 [18–28] | <0.001 |
| Contrast volume, mL | 8 [6–10] | 6 [4–8] | 0.03 |
| Contrast volume/eGFR ratio | 0.18 [0.13–0.25] | 0.25 [0.17–0.34] | 0.09 |
| Technical success | 35 (100.0) | 15 (100.0) | — |
| Major vascular complication | 1 (2.9) | 0 (0.0) | 0.51 |
| Major or life-threatening bleeding | 1 (2.9) | 0 (0.0) | 0.51 |
| Acute kidney injury, any stage | 1 (2.9) | 0 (0.0) | 0.51 |
| Acute kidney injury stage 2 or 3 | 0 (0.0) | 0 (0.0) | — |
| New renal replacement therapy | 0 (0.0) | 0 (0.0) | — |
| eGFR at discharge, mL/min/1.73 m² | 45 [37–54] | 25 [19–29] | <0.001 |
| eGFR at 30 days, mL/min/1.73 m² | 46 [38–55] | 26 [20–30] | <0.001 |
| Absolute eGFR change at 30 days, mL/min/1.73 m² | +3 [−1 to +6] | +2 [0 to +5] | 0.74 |
| Stable or improved renal function at 30 days | 31 (88.6) | 15 (100.0) | 0.18 |
| Relative eGFR decline >10% at 30 days | 4 (11.4) | 0 (0.0) | 0.18 |
| All-cause death at 30 days | 0 (0.0) | 0 (0.0) | — |
| Heart failure rehospitalization at 30 days | 1 (2.9) | 0 (0.0) | 0.51 |

Values are mean ± standard deviation, median [interquartile range], or n (%). eGFR, estimated glomerular filtration rate.

**Supplementary Table S4. Outcomes according to contrast volume category**

| **Variable** | **≤5 mL contrast (n = 13)** | **6–9 mL contrast (n = 22)** | **10 mL contrast (n = 15)** | **p value** |
| --- | --- | --- | --- | --- |
| Baseline eGFR, mL/min/1.73 m² | 31 [22–42] | 39 [30–48] | 42 [35–54] | 0.07 |
| Baseline creatinine, mg/dL | 1.86 [1.36–2.61] | 1.50 [1.20–2.02] | 1.38 [1.10–1.72] | 0.08 |
| Contrast volume/eGFR ratio | 0.13 [0.10–0.19] | 0.20 [0.15–0.29] | 0.25 [0.18–0.33] | 0.02 |
| Technical success | 13 (100.0) | 22 (100.0) | 15 (100.0) | — |
| Mean gradient at 30 days, mmHg | 8.5 ± 3.0 | 8.8 ± 3.4 | 8.7 ± 3.1 | 0.96 |
| Mild paravalvular regurgitation at 30 days | 4 (30.8) | 7 (31.8) | 4 (26.7) | 0.94 |
| Moderate/severe paravalvular regurgitation at 30 days | 0 (0.0) | 0 (0.0) | 0 (0.0) | — |
| Acute kidney injury, any stage | 0 (0.0) | 1 (4.5) | 0 (0.0) | 0.45 |
| New renal replacement therapy | 0 (0.0) | 0 (0.0) | 0 (0.0) | — |
| Relative eGFR decline >10% at 30 days | 1 (7.7) | 2 (9.1) | 1 (6.7) | 0.99 |
| Stable or improved renal function at 30 days | 12 (92.3) | 20 (90.9) | 14 (93.3) | 0.96 |
| All-cause death at 30 days | 0 (0.0) | 0 (0.0) | 0 (0.0) | — |

Values are mean ± standard deviation, median [interquartile range], or n (%). eGFR, estimated glomerular filtration rate.

**Supplementary Table S5. Renal outcomes according to contrast volume/eGFR ratio**

| **Variable** | **Contrast volume/eGFR ratio tertile 1 (n = 17)** | **Tertile 2 (n = 16)** | **Tertile 3 (n = 17)** | **p value** |
| --- | --- | --- | --- | --- |
| Contrast volume/eGFR ratio | 0.11 [0.08–0.14] | 0.21 [0.18–0.24] | 0.35 [0.30–0.47] | <0.001 |
| Baseline eGFR, mL/min/1.73 m² | 51 [45–60] | 38 [33–44] | 26 [20–33] | <0.001 |
| Contrast volume, mL | 6 [4–7] | 8 [7–9] | 9 [8–10] | <0.001 |
| Acute kidney injury, any stage | 0 (0.0) | 1 (6.3) | 0 (0.0) | 0.36 |
| Acute kidney injury stage 2 or 3 | 0 (0.0) | 0 (0.0) | 0 (0.0) | — |
| New renal replacement therapy | 0 (0.0) | 0 (0.0) | 0 (0.0) | — |
| Absolute eGFR change at discharge, mL/min/1.73 m² | +2 [−1 to +5] | +2 [−2 to +5] | +3 [0 to +6] | 0.64 |
| Absolute eGFR change at 30 days, mL/min/1.73 m² | +3 [−1 to +6] | +2 [−1 to +6] | +3 [0 to +7] | 0.71 |
| Relative eGFR decline >10% at 30 days | 1 (5.9) | 2 (12.5) | 1 (5.9) | 0.70 |
| Stable or improved renal function at 30 days | 16 (94.1) | 14 (87.5) | 16 (94.1) | 0.70 |

Values are median [interquartile range] or n (%). eGFR, estimated glomerular filtration rate.

**Supplementary Table S6. Outcomes according to MyVal size category**

| **Variable** | **Standard MyVal sizes (n = 28)** | **Intermediate MyVal sizes (n = 22)** | **p value** |
| --- | --- | --- | --- |
| Annular area, mm² | 464 ± 82 | 491 ± 70 | 0.22 |
| Contrast volume, mL | 8 [6–10] | 7 [5–10] | 0.36 |
| Technical success | 28 (100.0) | 22 (100.0) | — |
| Post-dilatation | 4 (14.3) | 3 (13.6) | 0.95 |
| Mean gradient at discharge, mmHg | 8.6 ± 3.1 | 8.1 ± 2.8 | 0.56 |
| Mean gradient at 30 days, mmHg | 8.9 ± 3.4 | 8.4 ± 3.0 | 0.59 |
| Aortic valve area at 30 days, cm² | 1.81 ± 0.34 | 1.86 ± 0.30 | 0.61 |
| No/trace paravalvular regurgitation at 30 days | 19 (67.9) | 16 (72.7) | 0.71 |
| Mild paravalvular regurgitation at 30 days | 9 (32.1) | 6 (27.3) | 0.71 |
| Moderate/severe paravalvular regurgitation at 30 days | 0 (0.0) | 0 (0.0) | — |
| New permanent pacemaker implantation | 2 (7.1) | 2 (9.1) | 0.80 |

Values are mean ± standard deviation, median [interquartile range], or n (%).

**Supplementary Table S7. Hemodynamic outcomes according to annular area**

| **Variable** | **Annular area <430 mm² (n = 16)** | **Annular area ≥430 mm² (n = 34)** | **p value** |
| --- | --- | --- | --- |
| Baseline mean gradient, mmHg | 47.1 ± 12.4 | 44.3 ± 11.5 | 0.43 |
| Baseline aortic valve area, cm² | 0.59 ± 0.12 | 0.70 ± 0.15 | 0.01 |
| Intermediate MyVal size used | 5 (31.3) | 17 (50.0) | 0.22 |
| Mean gradient at discharge, mmHg | 9.8 ± 3.4 | 7.8 ± 2.6 | 0.03 |
| Mean gradient at 30 days, mmHg | 10.2 ± 3.5 | 8.0 ± 2.8 | 0.02 |
| Aortic valve area at 30 days, cm² | 1.62 ± 0.24 | 1.93 ± 0.30 | <0.001 |
| Residual gradient ≥20 mmHg at 30 days | 0 (0.0) | 0 (0.0) | — |
| Mild paravalvular regurgitation at 30 days | 5 (31.3) | 10 (29.4) | 0.89 |
| Moderate/severe paravalvular regurgitation at 30 days | 0 (0.0) | 0 (0.0) | — |

Values are mean ± standard deviation or n (%).

**Supplementary Table S8. Chronological analysis of procedural performance**

| **Variable** | **First 25 patients** | **Last 25 patients** | **p value** |
| --- | --- | --- | --- |
| Contrast volume, mL | 9 [8–10] | 6 [5–8] | <0.001 |
| Contrast volume ≤5 mL | 1 (4.0) | 12 (48.0) | <0.001 |
| Contrast volume/eGFR ratio | 0.25 [0.18–0.35] | 0.16 [0.10–0.24] | 0.01 |
| Procedure time, min | 69 [58–84] | 57 [49–68] | 0.01 |
| Fluoroscopy time, min | 16.7 [13.5–21.0] | 12.9 [10.2–16.5] | 0.01 |
| Dose-area product, Gy·cm² | 50 [36–70] | 35 [24–52] | 0.03 |
| Technical success | 25 (100.0) | 25 (100.0) | — |
| Post-dilatation | 4 (16.0) | 3 (12.0) | 0.68 |
| Major vascular complication | 1 (4.0) | 0 (0.0) | 0.31 |
| Major or life-threatening bleeding | 1 (4.0) | 0 (0.0) | 0.31 |
| Acute kidney injury, any stage | 1 (4.0) | 0 (0.0) | 0.31 |
| New permanent pacemaker implantation | 2 (8.0) | 2 (8.0) | 1.00 |
| Device success at 30 days | 25 (100.0) | 25 (100.0) | — |

Values are median [interquartile range] or n (%).

**Supplementary Table S9. Exploratory multivariable analyses of contrast use, procedural efficiency, and 30-day renal-function change**

| **Outcome and covariate** | **Adjusted β** | **95% CI** | **p value** |
| --- | --- | --- | --- |
| **Model 1: Total contrast volume, mL** |  |  |  |
| Chronological case number, per 10-case increase | −0.74 | −1.08 to −0.40 | <0.001 |
| Baseline eGFR, per 10 mL/min/1.73 m² increase | +0.12 | −0.08 to +0.32 | 0.23 |
| Anatomical-complexity indicator | +0.83 | +0.12 to +1.54 | 0.023 |
| **Model 2: Procedure time, min** |  |  |  |
| Chronological case number, per 10-case increase | −4.6 | −7.3 to −1.9 | 0.001 |
| Anatomical-complexity indicator | +8.4 | +2.2 to +14.6 | 0.009 |
| Pre-dilatation | +6.7 | +1.1 to +12.3 | 0.020 |
| **Model 3: Absolute eGFR change at 30 days, mL/min/1.73 m²** |  |  |  |
| Baseline eGFR, per 10 mL/min/1.73 m² increase | −1.3 | −2.2 to −0.4 | 0.006 |
| Contrast volume/eGFR ratio | +0.8 | −2.6 to +4.2 | 0.64 |
| Hemoglobin decrease, per 1 g/dL | −1.1 | −2.0 to −0.2 | 0.018 |
